# Tools for Assessment of Country Preparedness for Public Health Emergencies: A Critical Review

**DOI:** 10.1017/dmp.2020.13

**Published:** 2021-08

**Authors:** Mariana Haeberer, Svetla Tsolova, Paul Riley, Rosa Cano-Portero, Ute Rexroth, Massimo Ciotti, Graham Fraser

**Affiliations:** Institute of Health Carlos III, Madrid; European Centre for Disease Prevention and Control, Stockholm; PERPHECT Consortium, Public Health England, Salisbury; Robert Koch Institute, Berlin

**Keywords:** assessment, emergency preparedness, Europe, health system, planning, public health, tool

## Abstract

Recent international communicable disease crises have highlighted the need for countries to assure their preparedness to respond effectively to public health emergencies. The objective of this study was to critically review existing tools to support a country’s assessment of its health emergency preparedness. We developed a framework to analyze the expected effectiveness and utility of these tools. Through mixed search strategies, we identified 12 tools with relevance to public health emergencies. There was considerable consensus concerning the critical preparedness system elements to be assessed, although their relative emphasis and means of assessment and measurement varied considerably. Several tools identified appeared to have reporting requirements as their primary aim, rather than primary utility for system self-assessment of the countries and states using the tool. Few tools attempted to give an account of their underlying evidence base. Only some tools were available in a user-friendly electronic modality or included quantitative measures to support the monitoring of system preparedness over time. We conclude there is still a need for improvement in tools available for assessment of country preparedness for public health emergencies, and for applied research to increase identification of system measures that are valid indicators of system response capability.

International communicable disease crises of recent years have highlighted the need for countries to assure their preparedness to respond effectively to public health emergencies.^1-3^ Further, implementation of the revised International Health Regulations (IHR), a 2013 decision of the European Parliament and Commission on cross border health threats, and concerns over the effectiveness of the initial response to the Ebola crisis, have led to calls for countries to assess and report on the effectiveness of their public health emergency preparedness and response organization and plans.^4-6^

Despite this, there is only limited knowledge of the methods by which countries assess the state of their preparedness for public health emergencies. There is also little consensus regarding the system elements that should be included in a preparedness system assessment and their validity as predictors of country responsiveness during an actual emergency.^7,8^ Available assessment tools were reviewed by Asch et al.^9^ and Nelson et al.^10^ in 2005 and 2007, respectively, expressing concerns over the validity of methodologies then in use. Since then, a number of assessment methodologies and tools have been developed, with varying relevance to public health emergency preparedness.

The objective of this study was to review the characteristics and utility of available tools and methodologies for assessment of countries’ public health emergency preparedness. The underlying purpose of this review was to guide development by the European Centre for Disease Prevention and Control (ECDC) of methodological concepts and tools to support public health emergency preparedness in European Union (EU)/European Economic Area (EEA) member states.

## METHODS

Existing tools were located through 3 lines of inquiry: (1) MEDLINE search of peer-reviewed literature, (2) online search of gray literature in public health and civil defense websites, and (3) e-mail contact with ECDC designated emergency preparedness respondents for all EU/EEA member states, to identify nationally developed tools that had not been published elsewhere. Methods 1 and 2 were independently performed by 2 reviewers. The review was conducted between October and December 2014, with updates up to August 2018.

For the MEDLINE search, following a pilot exploration of the data sources and literature, all key words/MeSH terms that could be related with public health emergency preparedness assessment tools were included, and search filters were chosen to be non-restrictive in order to increase search sensitivity. These are given in Table 1. Inclusion criteria were (1) Period: 2000–2017; (2) Languages: reports published in English, Spanish, French, German, or Italian; (3) Category: humans; (4) Scope: subnational, national, or international; (5) Type of hazard: generic (ie, all-hazards approach), or pandemic influenza; and (6) Presence of a checklist, indicators, or measures to assess national public health emergency preparedness status. Civil protection emergency assessment tools were excluded if they did not include public health or health care aspects of the emergency response. The gray literature search included international and national public health and civil defense websites.

**TABLE 1:** MEDLINE and Gray Literature Search Strategies

Source	Search Strategy
**MEDLINE**	(“public health” [All Fields] OR “health system” [All Fields]) AND (“emergencies” [MeSH Terms] OR “emergencies” [All Fields] OR “emergency” [All Fields]) OR (“disasters” [MeSH Terms] OR “disasters” [All Fields] OR “disaster” [All Fields]) OR (“pandemics” [MeSH Terms] OR “pandemics” [All Fields] OR “pandemic” [All Fields]) AND (“planning” [All Fields] OR “preparedness” [All Fields] OR “response” [All Fields]) AND (“assessment” [Journal] OR “assessment” [All Fields]) OR (“measurement (Mahwah N J)” [Journal] OR “measurement” [All Fields]) OR tool [All Fields] OR toolkit [All Fields] OR (“checklist” [MeSH Terms] OR “checklist” [All Fields]) OR standard [All Fields]) AND (“2000/01/01” [PDAT]: “2017/08/29” [PDAT]) AND “humans” [MeSH Terms])
**Public Health, Security, Civil Defense, and other emergency management-related institutional websites**	(“public health” OR “health system”) AND (emergency OR disaster OR pandemic) AND (planning OR preparedness OR response) AND (evaluation OR assessment OR measurement OR tool OR toolkit OR checklist OR standard) Websites: World Health Organization; European Centre for Disease Prevention and Control; European Commission; European Parliament; United Nations; Organization for Security and Cooperation in Europe; United States Centers for Disease Control and Prevention; Ministries of Health and of Security and Civil Defence (European Union and European Economic Area countries; United States; New Zealand; Australia; Canada)

A framework to review and compare the identified tools was developed by the investigators, drawing substantively on criteria developed by Nelson et al.^10^ and Asch et al.^9^ Complementary indicators were extracted from these publications and combined in a single analytical framework, together with 2 further indicators developed by the authors (“completeness,” “main advantages”) (Table 2).

**TABLE 2:** Evaluation Framework

Criteria	Description
**Completeness**	Degree of coverage of the main areas identified in the analysis. The coverage (yes/no) of a certain area was assessed only considering the inclusion of that area in at least 1 indicator or question.
**Clarity of measurement parameters**	Extent to which the methods for measurement of the indicators, actions, or structures were explicitly stated in the tool.
**Validity and specificity of scope and measurement parameters**	Extent to which the tool measured the attributes that this study sought to measure regarding scope (national and subnational focus, European Union countries) and parameters. Presence of validation specifications.
**Evidence based**	Extent to which the observational or experimental evidence was clearly provided for the indicated actions or capacities.
**Feasibility**	Extent to which the measures were user friendly and did not impose excessive burdens. This was assessed on author’s judgment when they have tried to use and/or complete the different tools.
**Utility**	Extent to which the tool supported decisions related to improvement, accountability, and other functions. Improvement-oriented assessments are usually aimed at internal audiences. Accountability-oriented assessments are usually aimed at external stakeholders.^10^ In this case, utility was linked to the actual use of the tool output (eg, list of tasks to perform vs external evaluation measures) and the methodology used (eg, qualitative vs quantitative).
**Specification of an accountable entity**	Extent to which the tool identified an individual or group of the evaluated institution as responsible for completion of each component indicator.
**Main advantages**	Characteristics of the tool that could be useful for a European tool development.

Modified from Asch et al.^9^ and Nelson et al.^10^

## RESULTS

A total of 1459 peer-reviewed articles were retrieved and screened for eligibility. Only 2 assessment tools meeting the quality criteria were identified through a peer-review literature search: United States National Health Security Preparedness Index (NHSPI™)^11^ and Harvard School of Public Health Emergency Preparedness Exercise Evaluation Tool^a^ (H-EPREP).^12^ Another 9 tools were identified from the search of public health and civil defense websites: European Commission technical guidance on generic preparedness planning for public health emergencies^13^ and template for providing information on preparedness and response planning in relation to serious cross-border threats to health^14^; World Health Organization (WHO) questionnaire for monitoring progress in the implementation of IHR Core Capacities In States Parties^15^ and Joint External Evaluation Tool (JEE)^16^; Toolkit for assessing health system capacity for crisis management of the WHO Regional Office for Europe^17^; Joint European Pandemic Preparedness Self-Assessment Indicators^18^; National Health Services England Core Standards for Emergency Preparedness, Resilience, and Response (EPRR)^19^; New Zealand Civil Defence and Emergency Management (CDEM) Capability Assessment Tool^20^; and United States Centers for Disease Control and Prevention (CDC) Public Health Preparedness Capabilities.^21^ One further tool was identified by an ECDC national respondent: the EpiSouth National Generic Emergency Preparedness Plans^a^ (E-EPREP).^22^

The 12 tools identified are summarized in Table 3. All were published between 2009 and 2016. Seven of the 12 tools were developed by international authorities or organizations.^13-18,22^ The other 5 were country-specific: from England,^19^ New Zealand,^20^ and the United States.^11,12,21^ Some of the tools developed by international organizations had a primary focus on voluntary country level implementation.^13,17,18,22^ Others had an apparent primary rationale of required or recommended reporting under international or European regulations.^14-16^

**TABLE 3:** Summary of Tools Identified for Comparison

Country/Organization	Name of the Tool
European Commission (EC), 2005, 2011^13^	EC Strategy for Generic Preparedness Planning. Technical guidance on generic preparedness planning for public health emergencies.
European Commission, 2014^14^	Commission Implementing Decision of 25 July 2014 implementing Decision No 1082/2013/EU of the European Parliament and of the Council with regard to the template for providing the information on preparedness and response planning in relation to serious cross-border threats to health.
World Health Organization (WHO), 2014^15^	International Health Regulation (IHR) core capacity monitoring framework: questionnaire for monitoring progress in the implementation of IHR Core Capacities In States Parties.
WHO, 2016^16^	Joint External Evaluation (JEE) tool
WHO Regional Office for Europe, 2012^17^	Toolkit for assessing health-system capacity for crisis management
WHO Regional Office for Europe, European Centre for Disease Prevention and Control (ECDC) and EC, 2010^18^	Joint European Pandemic Preparedness Self-Assessment Indicators
England: National Health Services (NHS), 2014^19^	NHS England Core Standards for Emergency Preparedness, Resilience and Response (EPRR)
New Zealand: Civil Defence and Emergency Management (CDEM), 2014^20^	CDEM Capability Assessment Tool
United States (US): Centers for Disease Control and Prevention (CDC), 2011^21^	US CDC Public Health Preparedness Capabilities: National Standards for State and Local Planning
US: Harvard School of Public Health, 2015^12^	Harvard School of Public Health Preparedness and Emergency Response Research Center. Emergency Preparedness Exercise Evaluation Tool (H-EPREP).
US: Association of State and Territorial Health Officials (ASTHO) and CDC, 2013^11^	US National Health Security Preparedness Index (NHSPI™)
EpiSouth Network, 2013^22^	National Generic Emergency Preparedness Plans (E-EPREP)

Appraisal of the tools according to the evaluation framework is summarized in Table 4. All tools identified had governmental or institutional authorities as the principal target audience and specified an accountable entity, but with varying degrees of detail. Although all tools had public health emergencies as a primary focus, they varied in their relative emphasis on various aspects of emergency preparedness, including health system resilience and the wider civil emergency protection function. All except 1^18^ took an all-hazards approach, although they mainly focused on communicable (infectious) disease emergencies with some additional sections for other types of public health hazard such as chemical or radiological events.

**TABLE 4:** Evaluation Framework and Comparison of Identified Tools

Name of the Tool	EC Technical Guidance on Generic Preparedness Planning for Public Health Emergencies^13^	EC Template for Reporting on Decision No 1082/2013/EU^14^	WHO Questionnaire for Monitoring IHR Core Capacities in States Parties^15^	WHO Joint External Evaluation Tool^16^
**General Description**	Checklists (tasks for every outcome expected) outlining the essential minimum requirements for public health emergency preparedness	Checklist (questions and indicators) assessing four areas for managing serious cross-border threats	Checklist with 244 global indicators for monitoring the development and maintenance of international health regulations (IHR) 13 core capacities	Checklist with 48 global indicators for regular external evaluations of a country’s IHR capacity (~every 5 years). Voluntary country participation
**Target Audience**	European Union (EU) Member States government authorities, European Commission (EC) and Agencies	EU Member States government authorities	World Health Organization (WHO) Member States government authorities responsible for implementing IHR	WHO Member States government authorities responsible for implementing IHR
**Scope**	International and national	International and national	International and national	International and national
**Type of Hazard**	All hazards	All hazards	All hazards	All hazards
**Completeness** (criteria based on Table 4)	Incomplete: recovery, community preparedness, and funding areas not covered	Incomplete: focus only on IHR core capacities monitoring, interoperability, business continuity management, and evaluation of plans	Incomplete: recovery, business continuity management, community preparedness, and other areas not covered	Incomplete: recovery, business continuity management, community preparedness, and other areas not covered
**Clarity of Measurement Parameters**	Clear description of indicators Binary (yes/no) answer system	Clear description of questions and indicators Open or (yes, no, not known) answer system	Clear description of indicators Open or (yes, no, not known) answer system	Clear description of indicators Every indicator has various levels of capacity with color coding scores of 1–5. For each indicator, a country will receive a single score
**Validity and Specificity of Scope and Measurement Parameters**	National focus included High specificity to EU Very high level of detail, no validation described No quantification	National focus High specificity to EU High level of detail in some indicators but many open questions, no validation described No quantification	National focus Low specificity to EU Good level of detail and specificity of questions, no validation described No quantification	National focus Low specificity to EU High level of detail and specificity of questions, external validation Simple quantification
**Evidence Based**	Expert-consensus	Expert-consensus	Expert-consensus	Expert-consensus. Also incorporates content and lessons learned from tested external assessment tools and processes of other multilateral and multi-sectoral initiatives
**Feasibility**	Limited by its comprehensiveness (covers a large number of dimensions and themes) and by its format (paper based, plain text)	Limited by its format (paper based, plain text, several tables with different formats, different type of answers mixed)	Although paper based, it is clear and simple	Although paper based and comprehensive, it is clear and color score makes it straightforward
**Utility**	Includes qualitative self-rating measures List of tasks	Include qualitative self-rating measures List for inter-sectoral collaboration	Includes qualitative self-rating measures	Includes qualitative self-rating and external evaluation measures
**Accountable Entity Specified**	Yes	Yes	Yes	Yes
**Main Advantages**	Comprehensive scope: almost all dimensions covered Focus on EU countries Useful list of tasks	Includes Interoperability, business continuity management, and evaluation of plans Focus in EU countries Mandatory requirement of the EC	International standards (wide consensus) Comprehensive scope Clear description of indicators Mandatory requirement of the WHO	International standards (wide consensus) Comprehensive scope Clear description of indicators Voluntary requirement of the WHO: first time will be a baseline measurement of the country’s capacity and capabilities. Subsequent evaluations will identify progress made and ensure any improvements in capacity are sustained
**Name of the Tool**	**WHO Toolkit for Assessing Health System Capacity for Crisis Management^17^**	**Joint European Pandemic Preparedness Self-Assessment Indicators^18^**	**NHS England Core Standards for EPRR^19^**	**CDEM Capability Assessment Tool^20^**
**General Description**	Checklist with 51 essential attributes, corresponding to 16 key components of each of the 6 WHO health system framework functions blocks	Checklist with 20 goals and corresponding key indicators for pandemic influenza preparedness	Checklist with 37 generic and 13 specific (hazardous materials and chemical, biological, radiological, and nuclear events) response core standards	Index based on key performance indicators and measures organized in 6 sections: 4 based on goals of the National Civil Defence and Emergency Management (CDEM) strategy and 2 “enabler” sections
**Target Audience**	EU Member States Coordination Group (public health and other institutions) responsible for health sector crisis management	EU Member States person(s) responsible for the national pandemic planning and preparedness	National Health System (NHS) organizations and providers of NHS funded care system	New Zealand public health agencies and CDEM groups
**Scope**	National	National	National and subnational	National and subnational
**Type of Hazard**	All hazards	Influenza	All hazards	All hazards
**Completeness** (criteria based on Table 4)	Incomplete: recovery, business continuity management, community preparedness, and evaluation not covered	Incomplete: not all hazards and risk based approach, several dimensions not covered	Almost complete: recovery and health system operational response not fully covered	Almost complete: health system operational response not fully covered
**Clarity of Measurement Parameters**	Clear description of indicators Traffic lights system methodology (yes, partial, no)	Clear description of indicators (yes, partial, no) answer system	Clear description of indicators Traffic lights system methodology (yes, partial, no)	Very clear description of indicators Scoring system methodology
**Validity and Specificity of Scope and Measurement Parameters**	National focus Low specificity to EU Good level of detail and specificity of indicators, no validation described No quantification	National focus High specificity to EU Focus on one disease Good level of detail and specificity of indicators, no validation described No quantification	Focus on NHS system High specificity to subnational sector Good level of detail of indicators and assurance mechanisms, high specificity but no validation described No quantification	High specificity to subnational sector Very high level of detail of indicators but no validation described Quantitative and comparative assessment (index)
**Evidence Based**	Expert-consensus	Expert-consensus	Not described	Not described
**Feasibility**	Although paper based, it is clear, simple, and color score makes it straightforward and attractive	Although paper based and plain text, it is short, clear, and simple	Excel spreadsheet, so it’s easy to use but difficult to understand at first sight because only plain text is provided	It is an automatized Excel spreadsheet, and although at first sight seems complex, it is very simple with a very attractive format. Automatized results are very useful.
**Utility**	Includes qualitative self-rating measures	Includes qualitative self-rating measures	Includes qualitative self-rating measures	Includes quantitative self-rating measures Allows comparability between organizations (high transparency)
**Accountable Entity Specified**	Yes	Yes	Yes	Yes
**Main Advantages**	International standards Comprehensive scope Clear description of indicators and traffic lights system methodology	Focus in EU countries	User-friendly (Excel format) High specificity to subnational sector	Risk based and integral approach: Includes all hazards, all phases of emergency (preparedness, prevention, response, recovery and evaluation), community preparedness, integration of national and local services, business continuity management, and interoperability/inter-sectoral collaboration User-friendly (automatized Excel format) High specificity to subnational sector Quantitative and comparative assessment (index) allows to monitor and to show graphic results Scoring system allows to weight indicators according to national or regional priorities
**Name of the Tool**	**CDC Public Health Preparedness Capabilities^21^**	**Emergency Preparedness Exercise Evaluation Tool (H-EPREP)^12^**	**United States NHSPI^11^**	**EpiSouth Network E-EPREP^22^**
**General Description**	Checklist with 15 public health emergency preparedness capabilities organized in 6 categories. Each capability includes a list of functions, performance measures, tasks, and resource considerations	Exercise evaluation tool: combination of checklists and rating scales to produce quantifiable representations of 160 tasks and 500 related actions to assess performance	Index based on 128 indicators, organized in 5 domains and 14 subdomains measuring key areas of public health emergency preparedness. National results are calculated by averaging the 50 states	List of tasks to perform for developing national generic plans
**Target Audience**	United States (US) state and local public health departments	US state and local public health organizations	US policy-makers, practitioners, researchers, and communicators	Health sector of Mediterranean Basin EU and non-EU countries
**Scope**	National and subnational	National and subnational	National and subnational	National
**Type of Hazard**	All hazards	All hazards	All hazards	All hazards
**Completeness** (criteria based on Table 4)	Incomplete: governance, legal framework, and other areas not covered	Incomplete: governance, legal framework, and other areas not covered	Incomplete: governance, legal framework, funding, and other areas not covered	Almost complete
**Clarity of Measurement Parameters**	Clear description of the few indicators included	Very clear description of performance measures Binary (yes/no) answer system for specific actions and 1-10 scale for general task Overall performance	Very clear description of indicators Complex scoring system methodology	Very clear description of expected outcomes
**Validity and Specificity of Scope and Measurement Parameters**	Focus on US needs High specificity to subnational sector Very high level of detail of the few indicators but no validation described Limited qualitative assessment (no quantification)	Focus on US needs High specificity to subnational sector Very high level of detail Validation method described: tested for reliability, usability, and validity by independent evaluators during multiple exercises Qualitative and simple quantitative assessment	Focus on US needs and data availability High specificity to subnational sector Very high level of detail and specificity of indicators Quantitative and comparative assessment Validation problem: indicators chosen favors readily collectable measures	National focus High level of detail of tasks No quantification
**Evidence Based**	Systematic approach: based on evidence-informed documents, applicable preparedness literature and subject matter expertise gathered from across the federal government and the state and local practice community	Systematic approach: based on lessons learned from discussions with expert practitioners, from review of literature, and available tools	Target measure values ideally came from scientific study and practice, but only a few targets could be identified from the literature. Where literature and scientific data did not exist, target values were defined as those of the highest performing states	Not described
**Feasibility**	Although paper based, it is a clear and relatively short list of tasks	It is an online interactive tool with Excel outputs, very easy to use	It is an online tool with data query functionalities, very easy to use	Available as both a descriptive tool (limited feasibility because it is paper based and very comprehensive) and as online interactive tool (easier to use)
**Utility**	List of tasks Includes some qualitative self-rating measures	Includes qualitative and quantitative measures for assessing US Public Health Preparedness Capabilities Standardized but can be customized	It is not a self-assessment Includes quantitative measures Allows comparability between states (high transparency)	List of tasks Includes some qualitative self-rating measures
**Accountable Entity**	Not applicable	Yes	Yes	Yes
**Main Advantages**	Includes community preparedness Useful description of tasks in order to accomplish capabilities functions	Very user-friendly: online database of exercise evaluation measures that allows to generate customized exercise evaluation forms, store, and send them to multiple evaluators via e-mail, and generate basic reports Validation assessment described	Comprehensive scope User-friendly Quantitative and comparative assessment but it is made centrally by a large committee representing more than 25 organizations (it is not a self-assessment)	Includes interoperability/inter-sectoral collaboration Useful description of tasks User-friendly (online version) Possibilities for ongoing updating and revisions

The key assessment areas included in each of the tools are outlined in Table 5. Some areas were common to nearly all tools, with varying degrees of detail and methodological approaches, for example, interoperability and inter-sectoral collaboration, crisis management and operations, planning, communication and information systems, and human resources and capability development. Other assessment areas were addressed less frequently, for example, recovery, community preparedness, cross-border issues, or ethical aspects.

**TABLE 5:** Comparison of Key Areas Addressed in Identified Tools

Key Areas	EC ^13^	EC ^14^	WHO ^15^	WHO ^16^	WHO^17^	WHO ^18^	NHS ^19^	CDEM ^20^	CDC ^21^	H-EPREP^12^	NHSPI ^11^	E-EPREP ^22^
Health crisis management and principles of operation	X	-	X	X	X	X	X	X	X	X	X	X
Health sector incident management and hospital preparedness	X	-	X	X	X	X	-	-	X	X	X	X
Recovery planning and management	-	-	-	-	-	-	-	X	X	X	X	X
Evaluation of response	X	X	X	X	-	-	X	X	X	X	X	X
Community resilience, preparedness, and recovery	-	-	-	-	-	-	X	X	X	X	X	X
Governance	X	-	X	X	X	X	X	X	-	-	-	X
Management and testing of plans	X	-	X	X	X	X	X	X	X	X	X	X
Legal framework	X	-	X	X	X	X	X	X	-	-	-	X
Ethical considerations	X	-	-	-	-	X	-	-	-	-	-	-
Funding	-	-	X	X	X	X	X	X	X	X	X	X
Business continuity management	X	X	-	-	X	X	X	X	X	X	X	X
Communication systems and management	X	-	X	X	X	X	X	X	X	X	X	X
Information systems and management	X	-	X	X	X	X	X	X	X	X	X	X
Scientific/evidence-based advise	X	-	-	X	X	-	X	X	X	X	-	-
Human resources and capability development	X	-	X	X	X	X	X	X	X	X	X	X
Interoperability and Inter-sectoral collaboration	X	X	X	X	X	X	X	X	X	X	X	X
European Union level considerations	X	X	-	-	-	-	-	-	-	-	-	**-**

Most tools identified provided little or no information on the criteria or decision processes used to identify the measures included in them, or the evidential approach taken for their development. Exceptions included the JEE,^16^ CDC,^21^ H-EPREP,^12^ and NHSPI^11^ tools. In most cases, the development of preparedness standards appeared to be based primarily on consultations with groups of experts. Only a minority of tools attempted to describe a conceptual and strategic framework underlying their design.^11,12,21^ The Civil Defence and Emergency Management Tool^20^ from New Zealand had the most comprehensive, logical, and updated framework, consistent with current concepts of health emergency preparedness.^23,28^

Most of the selected tools had clear measurement parameters, with different methodological formats and complexity. These varied from a detailed list of tasks^13-15,21,22^ to simple qualitative scales,^12,16-19^ through to more complex scoring systems.^11,20^ Four of the tools included a quantitative element: JEE,^16^ CDEM,^20^ H-EPREP,^12^ and NHSPI.^11^ The CDEM tool had a scoring system with weighted indicators that can be customized according to national or regional priorities.^20^

Seven of the tools were paper-based only, with no electronic informatics to facilitate use.^13-18,21^ Two were presented for use as an Excel file (NHS^19^ and CDEM^20^), and 3 had online modalities (H-EPREP,^12^ NHSPI,^11^ and E-EPREP^22^). Two allowed a degree of customization by the user: CDEM^20^ and H-EPREP.^12^ H-EPREP^12^ was an exception, providing for the generation of customized exercise evaluation forms, storage, transmission to multiple evaluators by e-mail, and generation of basic reports.

## DISCUSSION

The use of systematic methods and tools for system assessment should have substantive benefits for the preparedness of countries for public health emergencies. The tool infrastructure should in itself have symbolic value to help communicate a coherent view of the emergency preparedness system to all participants. This should cover all of the elements critical to ensuring an effective response, including effective collaboration across sectors and between countries in responding to cross-border events. The systematic assessment of these elements should enable gaps and weaknesses to be proactively identified and addressed. To achieve this, tools should include assessment items, which are valid indicators of actual performance in an emergency. They should be available in user-friendly electronic modalities and include quantitative elements to support the monitoring of system preparedness over time and voluntary benchmarking with others, to promote learning and system improvement.

We have identified 12 presently available tools to support assessment of country preparedness for public health emergencies. Most tools were found through national and international websites, and it is possible that more may have been identified through gray literature in languages other than those included in this search, at subnational level, and sources such as postgraduate theses.

Few of the identified tools meet all of the above requirements. We acknowledge a potential limitation of our appraisal in that the tools were evaluated as a desktop exercise based on *a priori* criteria; however, the evidence base from user experience of the presently available tools is almost non-existent.

Our review suggests some possible contributing perspectives on this present situation. Available tools appear to have been developed with somewhat different primary aims and methodological approaches. Most tools developed by international agencies and 1 in the United States appeared to focus primarily on standard reporting requirements to which countries and states are subject. Exceptions included the self-evaluation checklists developed by the European Commission^13^ and the WHO Regional Office for Europe,^17^ which appear to be designed explicitly for country use. Tools developed by national authorities provide a primary focus on the evaluation needs of the country but may not extrapolate well for use by others, given country-specific characteristics of health and public health emergency response systems. Further, country-level tools may have less utility for subnational (regional, local) jurisdictions, and viceversa.

Country preparedness evaluations need to assess not only plans and capacities, but also system capabilities for effective response to actual emergencies. Several tools relied heavily on input data relating to system capacities and resources; while information concerning these is often readily available, it may be only indirectly predictive of the capability to respond to an emergency. Nelson et al. observed in 2007 that the few tools then available to assess preparedness status tended to focus on capacities, and little evidence existed that linked specific structures with the ability to execute effective response processes, noting that “structural measures may not be valid indicators of preparedness.”^10^ In reporting on a review of national influenza pandemic preparedness plans in the EU in 2012, Nicoll noted that some national authorities had ceased further preparedness development after producing written plans and had neither developed operational aspects nor tried to assess whether they would work in practice.^24^ The present study suggests only modest advance in this respect; among the identified tools, only the CDEM,^20^ H-EPREP,^12^ and JEE^16^ tools included significant consideration of system capabilities, as well as capacities.

The evidence base linking preparedness capacities and capabilities to health outcomes remain weak.^7,10^ Asch et al. noted in 2005 that most instruments for assessing public health emergency preparedness relied excessively on subjective or structural measures and lacked a scientific evidence base.^9^ Previous literature reviews have found that the majority of journal articles were commentaries and anecdotal case studies, based on qualitative analyses,^8,25-27^ a situation unchanged in our present literature search in support of this critical tools analysis (to be reported separately). One systematic review concluded that most studies lacked a rigorous design, raising questions about the validity of the results.^7^ It appears that more and better quality research into public health emergency management is needed for the development of useful assessment tools, and the validity of presently assessed system elements as predictive of actual response capability remains largely unverified. This is also the conclusion of the developers of other tools, which attempted to provide some evidence-based approach, who ended up relying mainly on lessons learned documents^11,12,16,21^ (see Table 4). A focus of future research should include the comparison of preparedness system *a priori* assessment scores and the actual system performance outcomes in real-life incidents and emergencies.

As the tools reviewed did not have a documented strong evidence base, there was only partial consensus on the system elements critical for public health emergency preparedness, and how they may be assessed or measured. Although some system areas were common to most tools, there was significant diversity in the system elements included and their emphasis across the tools reviewed, and in the indicators or standards used to measure their effective presence. “The problem lies not in the absence of standards *per se*, but in the multiplicity of overlapping (and sometimes conflicting) standards.”^10,28^

One issue underlying indicator development appears as differing preferences for standardizing all system measures, or leaving countries’ flexibility to modify, add, or delete them. Some authors have recommended standardization of all assessment measures^9,29^ in order to facilitate comparisons, either to a “gold standard” or between countries. However, some emergency response leaders consider that this is less useful than a flexible country-specific tool, given different country administrative structures and health care systems. Respondents to the EU pandemic influenza preparedness review in 2009 considered that “instead of [standardized] indicators, it would be more useful to develop a tool describing the main areas for consideration in pandemic influenza preparedness planning. Each country may then add its own criteria, indicators or outcomes for determining whether something is in place.”^23^ This choice, in turn, appears to also reflect divergent views on the perceived value of sharing country information and benchmarking with others. In the same review, “a number of member states made it clear to the ECDC that the country specific results should only be known to the country […] and that specifically there would be no ‘league tables.’”^23^

Few tools were available in user-friendly, electronic modalities that could facilitate data gathering, analysis, and dissemination and discussion of results by participants and stakeholders. H-EPREP^12^ was an exception, as it also allowed the generation of customized exercise evaluation forms, storage, transmission to multiple evaluators by e-mail, and generation of basic reports. Developers should therefore be encouraged to produce assessment tools in more user-friendly modalities. Inclusion of quantitative scoring systems usefully support the monitoring of progress in the development of a country’s public health emergency preparedness over time. Such quantitative scoring systems can also facilitate voluntary benchmarking with other countries. However, few tools included this feature. Only 2 tools had been published in a manner accessible to a conventional literature search^28,30^; most were available only through the websites of the organizations that developed them.

## CONCLUSIONS

Methods and processes for assessment of country systems are an integral part of a holistic approach to assuring country emergency preparedness, including simulation exercises, after action reports and peer reviews.^31^ We conclude, however, that few of the existing tools satisfy all or most of the requirements for utility and effectiveness discussed previously. There is a continuing need for further improvement in tools available for countries’ assessment of their preparedness for public health emergencies. Existing tools could be revised with critical review of the validity of their assessment elements and indicators, and availability in more user-friendly electronic format with analytical and reporting modalities. New tools could be developed *de novo* at country and supranational level based on both a country’s needs and best available evidence relating to the validity of its assessment elements and indicators.

The paucity of applied emergency response systems research remains a significant impediment to achieving these improvements. In particular, the elements of the preparedness system that are valid indicators of actual response capability remain poorly understood. Reporting and critical review of user experience of all of the different means of evaluating country preparedness should contribute to this goal.^31^

## Footnotes

a Since both tool acronyms are *EPREP*, we have specified the Harvard tool as *H-EPREP* and EpiSouth tool as *E-EPREP*.
